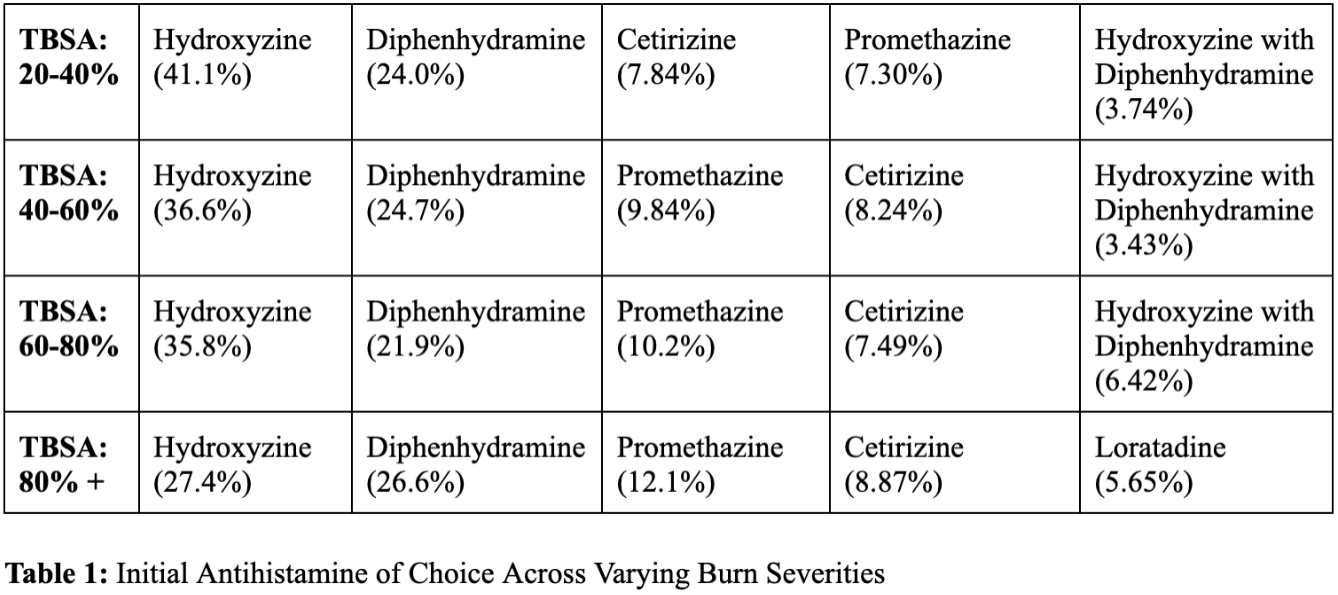# 931 Prescription Analysis of Antihistamines’ Use in Patients with Moderate to Severe Burns

**DOI:** 10.1093/jbcr/iraf019.462

**Published:** 2025-04-01

**Authors:** Abbas Karim, Nizam Karim, Suhaib Shah, Rashid Syed, Zain Akbar, Farhad Marzook, Juquan Song, George Golovko, Steven Wolf, Amina El Ayadi

**Affiliations:** University of Texas Medical Branch, John Sealy School of Medicine; University of Texas Medical Branch, John Sealy School of Medicine; University of Texas Medical Branch; University of Texas Medical Branch; University of Texas Medical Branch; University of Texas Medical Branch; University of Texas Medical Branch; University of Texas Medical Branch; University of Texas Medical Branch; University of Texas Medical Branch

## Abstract

**Introduction:**

Post-burn pruritus (PBP) significantly affects patients with moderate to severe burns (≥20% TBSA), causing discomfort and hindering recovery. Antihistamines, which block H1 receptors, are commonly prescribed for PBP, but large-scale studies on their usage patterns are limited. This study evaluates antihistamine prescription trends and provider practices for PBP across varying burn severities.

**Methods:**

We performed a treatment pathways analysis using TriNetX, a global, federated, deidentified database. A cohort of patients with burns ≥20% TBSA from the past 20 years (2004–2024) who developed pruritus was identified and stratified into four cohorts: 20-40%, 40-60%, 60-80%, and >80% TBSA. The analysis evaluated trends in antihistamine prescriptions, including the proportion treated with antihistamines, types prescribed, and median times to treatment initiation, duration, and therapy switching.

**Results:**

The total sample included 2,754 patients (20–40% TBSA, n=1,712; 40–60%, n=613; 60–80%, n=253; >80%, n=176). Over 70% of the total sample received antihistamines for pruritus management. Selection practices of antihistamines for PBP are characterized by a reliance on hydroxyzine as first-line therapy, followed by diphenhydramine. The average of the median times to treatment, on treatment, and before switching to another antihistamine, for the initial antihistamine of choice across all 4 cohorts, was 16.75 days, 90.25 days, and 27.75 days, respectively.

**Conclusions:**

Antihistamines, particularly hydroxyzine, are heavily relied upon for managing PBP in moderate to severe burns. Delays in treatment initiation indicate patients may remain symptomatic during critical recovery periods, impacting quality of life. The extended treatment durations and frequent switching indicate initial treatments may be suboptimal, necessitating more effective and timely therapeutic strategies, as well as potentially incorporating complementary therapies.

**Applicability of Research to Practice:**

These findings emphasize the importance of vigilant post-burn monitoring, earlier antihistamine initiation, and the development of standardized treatment protocols for PBP. Frequent therapy switching and prolonged therapy duration suggest current practices may benefit from enhanced symptom management tailored to burn severity.

**Funding for the Study:**

Database funding was provided by the National Center for Advancing Translational Sciences.